# Predicting Phenotypic Diversity and the Underlying Quantitative Molecular Transitions

**DOI:** 10.1371/journal.pcbi.1000354

**Published:** 2009-04-10

**Authors:** Claudiu A. Giurumescu, Paul W. Sternberg, Anand R. Asthagiri

**Affiliations:** 1 Division of Chemistry and Chemical Engineering, California Institute of Technology, Pasadena, California, United States of America; 2 Division of Biology, California Institute of Technology, Pasadena, California, United States of America; University of Illinois at Urbana-Champaign, United States of America

## Abstract

During development, signaling networks control the formation of multicellular patterns. To what extent quantitative fluctuations in these complex networks may affect multicellular phenotype remains unclear. Here, we describe a computational approach to predict and analyze the phenotypic diversity that is accessible to a developmental signaling network. Applying this framework to vulval development in *C. elegans*, we demonstrate that quantitative changes in the regulatory network can render ∼500 multicellular phenotypes. This phenotypic capacity is an order-of-magnitude below the theoretical upper limit for this system but yet is large enough to demonstrate that the system is not restricted to a select few outcomes. Using metrics to gauge the robustness of these phenotypes to parameter perturbations, we identify a select subset of novel phenotypes that are the most promising for experimental validation. In addition, our model calculations provide a layout of these phenotypes in network parameter space. Analyzing this landscape of multicellular phenotypes yielded two significant insights. First, we show that experimentally well-established mutant phenotypes may be rendered using non-canonical network perturbations. Second, we show that the predicted multicellular patterns include not only those observed in *C. elegans*, but also those occurring exclusively in other species of the *Caenorhabditis* genus. This result demonstrates that quantitative diversification of a common regulatory network is indeed demonstrably sufficient to generate the phenotypic differences observed across three major species within the *Caenorhabditis* genus. Using our computational framework, we systematically identify the quantitative changes that may have occurred in the regulatory network during the evolution of these species. Our model predictions show that significant phenotypic diversity may be sampled through quantitative variations in the regulatory network without overhauling the core network architecture. Furthermore, by comparing the predicted landscape of phenotypes to multicellular patterns that have been experimentally observed across multiple species, we systematically trace the quantitative regulatory changes that may have occurred during the evolution of the *Caenorhabditis* genus.

## Author Summary

The diversity of metazoan life forms that we experience today arose as multicellular systems continually sampled new phenotypes that withstood ever changing selective pressures. This phenotypic diversification is driven by variations in the underlying regulatory network that instructs cells to form multicellular patterns and structures. Here, we computationally construct the phenotypic diversity that may be accessible through quantitative tuning of the regulatory network that drives multicellular patterning during *C. elegans* vulval development. We show that significant phenotypic diversity may be sampled through quantitative variations without overhauling the core regulatory network architecture. Furthermore, by comparing the predicted landscape of phenotypes to multicellular patterns that have been experimentally observed across multiple species, we systematically deduce the quantitative molecular changes that may have transpired during the evolution of the *Caenorhabditis* genus.

## Introduction

During development, regulatory signaling networks instruct cell populations to form multicellular patterns and structures. To what extent perturbations in the quantitative performance of these networks may lead to phenotypic changes remains unclear. Experimental genetics studies typically uncover mutant phenotypes that emerge from extreme modes of perturbation (e.g., knockout or overexpression) [1],[2]. However, there is ample evidence that biological networks operate amidst quantitative fluctuations [3]–[6]. The sources of these quantitative perturbations include stochastic behavior, population heterogeneity, epigenetic effects and environmental changes.

The fundamental question then is how much phenotypic variation is possible by quantitative perturbations in network performance without wholesale changes to network topology. On the one hand, we may expect that the wild-type multicellular phenotype may be highly robust to quantitative variations. Indeed, computational analysis of the *Drosophila* segment polarity network demonstrated the robustness of the wild-type multicellular pattern to significant parameter changes [7]. This robustness may be a more pervasive property of developmental regulatory networks that allows their modular utilization in different multicellular geometries and developmental contexts [8]. On the other hand, for a given multicellular system, some degree of fragility in the regulatory network is essential for evolutionary diversification. New multicellular phenotypes must be accessible through modifications to the underlying regulatory network, providing avenues for sampling new phenotypes that may be more beneficial under different selective pressures.

The extent to which this phenotypic diversification must involve a topological overhaul of the regulatory network as opposed to quantitative changes to a fixed network topology remains unclear. Closely related species may have evolved by subtle, quantitative changes in network interactions rather than large-scale changes to network topology. Indeed, there is evidence for such “quantitative diversification” of phenotypes in the evolution of maize and finch beaks [9],[10]. However, analyzing extant species identifies only quantitative changes that have withstood selection and conceals the complete phenotypic diversity that a regulatory network can render. Meanwhile, experimentally reconstructing that diversity faces the challenge of systematically imposing quantitative regulatory perturbations *in vivo* and scoring the numerous phenotypes that would be generated.

**Figure 1:**
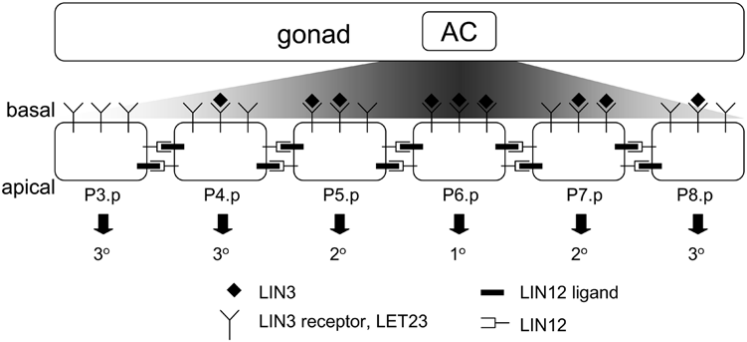
Wild-type patterning of *C. elegans* vulva. The anchor cell (AC) stimulates the vulva precursor cells Pn.p with LIN-3 in a graded manner. These cells laterally interact with their neighbors through the LIN-12 pathway. The crosstalk between LIN-3 and LIN-12 signaling results in the wild-type pattern of differentiation 3°3°2°1°2°3°. In the wild-type organism, the 1° vulval lineage generates progeny that forms the orifice and connects to the uterus, while the 2° vulval lineage generates progeny that form the vulval lips and connect to the body epidermis. The daughters of the 3° cells fuse to the surrounding syncytium and do not contribute to the vulval tissue.

Computational modeling has proven to be a useful tool for predicting multicellular patterns and morphology based on the underlying regulatory mechanisms [7], [11]–[18]. Thus, such models may provide an effective framework to explore the full diversity of phenotypes that is accessible through quantitative changes to a particular developmental regulatory network. Here, we develop a computational approach to analyze quantitatively the phenotypic diversity of *C. elegans* vulval development. The *C. elegans* vulva develops from an array of six precursor cells that commit to a spatial pattern of distinct fates (Figure 1) [19],[20]. We have described previously a mathematical model of the regulatory network that controls *C. elegans* vulval development and elucidated potential quantitative advantages of the biochemical coupling in this signaling network [21]. In this work, we extend this mathematical model of the signaling network to make predictions about the range of phenotypes that this network can render. We probed whether this developmental network is so robust to parameter changes that only a narrow set of multicellular phenotypes is possible. Or, can quantitative variations give rise to a broader range of phenotypes? In contrast to other recent models of *C. elegans* vulval development [12],[13], our model incorporates directly the underlying molecular mechanisms and the quantitative strength of these molecular regulatory pathways. Thus, it provides the necessary foundation for examining quantitative diversification of multicellular phenotype.

Our computational analysis reveals that a significant amount of phenotypic diversity is achievable through quantitative changes to the regulatory network. Thus, this developmental regulatory network is not “wired” to generate robustly only the wild-type phenotype. Furthermore, the phenotypes predicted by the model include not only those observed in *C. elegans*, but also those found exclusively in several closely-related species [22]. Thus, our model predictions validate the hypothesis that quantitative changes to a common regulatory network have occurred during the diversification of several species within the *Caenorhabditis* genus. Furthermore, by applying our modeling framework to analyze published experimental phenotypic data, we extract the quantitative regulatory differences that may have accrued during the evolution of three major species of the *Caenorhabditis* genus.

## Results and Discussion

### Gauging the phenotypic capacity of the vulval developmental network

**Figure 2:**
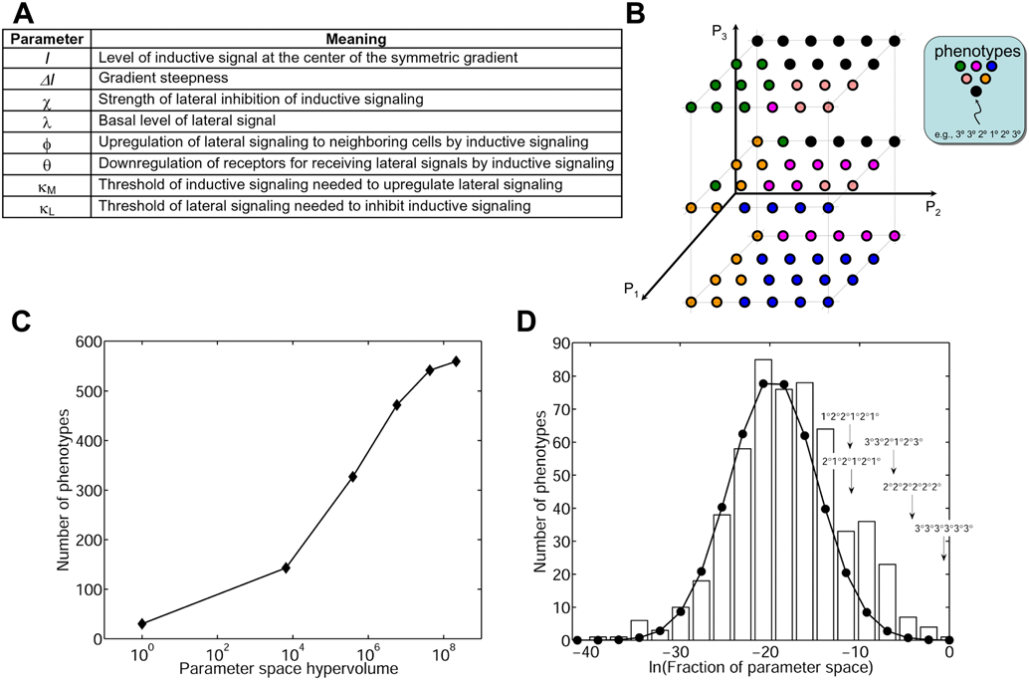
The predicted phenotypic diversity accessible to the vulval developmental network. (A) Model parameters. The model has eight dimensionless parameters associated with the various molecular interactions known to contribute to the specification of vulval precursor cells (see also Materials and Methods). (B) Schematic of the phenotype phase diagram. This diagram portrays a simplified, three-dimensional version of the 8-dimensional phenotype phase diagram. Each axis represents a model parameter. Each point in parameter space yields a specific multicellular phenotype, such as the wild-type (3°3°2°1°2°3°, black). (C) The total number of predicted phenotypes eventually saturates as the volume of the parameter space is expanded. (D) Distribution of parameter space occupancy (PSO). A histogram depicting the number of phenotypes (bars) occupying different fractions of the parameter space. This histogram is compared to a log-normal distribution (filled circles). The arrows indicate the PSO values of some experimentally observed phenotypes.

We sought to better understand how much phenotypic diversity a developmental regulatory network can produce through quantitative changes without altering the network architecture. To conduct this analysis, we started with our previously reported mathematical model of the regulatory network that controls vulval development in *C. elegans* [21]. This model uses ordinary differential equations to track the activity of two key signals in each precursor cell: MAP kinase and the lateral Notch signal (details are provided in Materials and Methods). The levels of these two signals are then used to predict the fate of each cell. The model consists of eight dimensionless parameters whose values influence the pattern of fate choices (Figure 2A). To determine the phenotypes that are accessible through quantitative modulation of the network, we allowed each parameter to vary across a broad range of physiological values (Materials and Methods). For each combination of parameter values, the multicellular phenotype was computed. In this manner, the multidimensional parameter space was divided into sub-regions associated with specific multicellular phenotypes (Figure 2B).

This phase diagram of phenotypes represents the predicted multicellular patterns that the vulval developmental network can produce. Extreme values along each parameter axis emulate the classical experimental scenario where specific molecular pathways are eliminated (e.g., knock out) or overexpressed. Away from these extremes, the phase diagram represents phenotypes that are predicted to occur when regulatory mechanisms are tuned quantitatively without wholesale changes to network topology. Thus, by counting the number of unique phenotypes that exist in this multidimensional parameter space, we sought to quantify the “phenotypic capacity” of the *C. elegans* vulval signaling network.

Our calculations show that the phenotypic capacity has an upper limit. That is, even as the parameter space is broadened, the number of distinct phenotypes saturates at approximately 560 multicellular patterns (Figure 2C). This result reveals that the developmental network is not constrained to a few outcomes. The wild type and a handful of well-studied mutant phenotypes by no means represent the phenotypic capacity of this system. Furthermore, in this six-cell system there are four fates possible to each cell (see Materials and Methods). Hence, the theoretical upper limit to the number of phenotypes is 4,096. Our model predicts that the molecular network constrains the system from accessing ∼85% of the theoretically possible phenotypes.

To better understand how the phenotypes are represented in parameter space, we determined the amount of parameter space associated with each phenotype (see Materials and Methods). Phenotypes that occur only at a few points in parameter space may be inaccessible experimentally, while their counterparts occupying a large fraction of parameter space may represent the more tangible outcomes. The distribution of Parameter Space Occupancy (PSO) resembles a log-normal distribution (μ = −19.60, σ = 4.90) with a slight positive skew (Figure 2D). On the low end of the distribution, our model predicts 19 phenotypes that are two standard deviations below the mean PSO (Table S4), and 9 of these phenotypes do not entail the mixed ‘m’ cell fate (Table S1). Consistent with this prediction, none of these predicted phenotypes are among the well-studied experimentally observed phenotypes. These highly unlikely outcomes reduce our evaluation of the overall phenotypic capacity of this system.

Meanwhile, on the other end of the distribution, a small subset of phenotypes occupies a disproportionately large portion of the parameter space (Figure 2D). Within the positive skew is the wild-type phenotype, consistent with a previous study that showed that the developmental segment polarity network robustly produces the wild-type multicellular pattern [7]. Extending beyond the wild-type phenotype, our model predicts an additional 33 phenotypes with PSO values that are two standard deviations above the mean (see Table S5 for a list of these phenotypes), 25 of which do not entail the mixed ‘m’ cell fate. These phenotypes are highly represented in parameter space and suggest that significant phenotypic diversity may be sampled by tuning quantitatively a common underlying regulatory network. In fact, consistent with model predictions, several of these 25 phenotypes have been observed in *C. elegans* genetics experiments [23],[24]. However, 10 of these 25 phenotypes have not been reported and are novel predicted phenotypes for future experimental validation.

**Figure 3:**
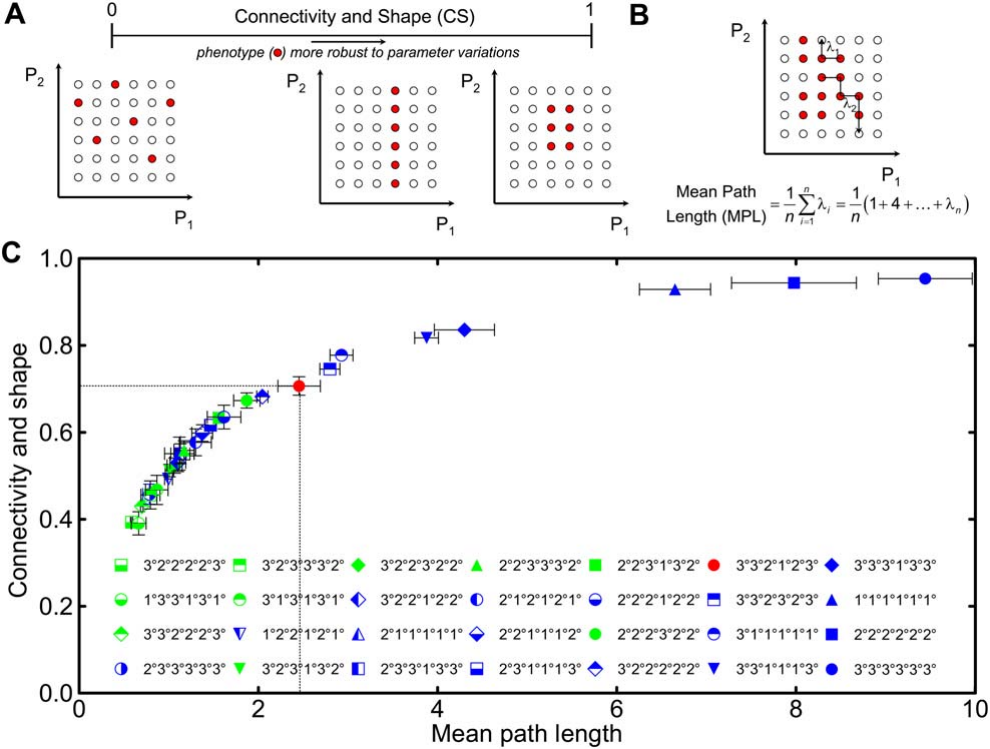
Robustness of phenotypes that are highly represented in parameter space. (A) Schematic of the Connectivity and Shape (CS) metric of phenotype robustness to parameter changes. The connectivity and shape metric measures the overall likelihood of staying in the current phenotype upon effecting a random unit-value parameter change from a randomly selected point in the phenotype. Isolated points in the parameter space do not contribute to the CS metric as unit-value parameter changes starting at such points would lead to exiting the phenotype. Interior points however are fully connected to neighbors and contribute the most to the CS metric. (B) Schematic of the Mean Path Length (MPL) metric of phenotype robustness to parameter changes. The mean path length metric measures the average number of unit-value parameter changes to exit the current phenotype. The larger the value, the more robust the phenotype to random parameter changes. (C) The CS and MPL of 26 phenotypes with the greatest PSO and 2 other experimentally observed phenotypes (2°1°2°1°2°1° and 1°2°2°1°2°1°). Experimentally observed phenotypes are denoted by blue symbols, while novel phenotypes that are not observed in *C. elegans* are denoted by green symbols. Dotted lines identify the value of CS and MPL for the wild-type phenotype. While MPL and CS follow each other monotonically, the CS is a better metric of the phenotype robustness at CS/MPL values lower than 0.5/1.0, while the MPL is a better metric at values higher than these thresholds.

To further evaluate these 10 novel phenotypes, we developed two metrics that provide additional insights into how phenotypes are distributed in parameter space. While the PSO metric quantifies what fraction of points in parameter space are associated with a particular phenotype, it does not report how these points are distributed in parameter space. One extreme is that the parameter points associated with a phenotype are disjointed and scattered throughout parameter space (Figure 3A). In this case, a perturbation in any parameter value would alter the phenotype, i.e. the phenotype would be highly fragile to parameter changes. The other extreme is that the parameter points are contiguous and clustered together into a subspace. In this scenario, the phenotype would be more robust to parameter variations. However, the level of robustness would depend on the shape of the phenotype subspace. A phenotype subspace that contains a high fraction of points at the “surface” (i.e., borders parameter points belonging to another phenotype) would be less robust than a phenotype where all its parameter points are tightly packed into a subspace with minimal exposure to other phenotypes.

To capture these aspects of how parameter points of a particular phenotype are distributed in parameter space, we developed a Connectivity and Shape (CS) metric (Materials and Methods). The value of the CS metric is bounded between 0 and 1 and represents the average likelihood that for any point in phenotype subspace, a unit change in any single parameter value maintains the phenotype (Figure 3A). Thus, a CS value of 0 would refer to a highly fragile phenotype whose points in parameter space are “isolated” or surrounded by other phenotypes. In contrast, a CS value of near 1 would refer to a highly robust phenotype for which most of the points in its parameter subspace are surrounded by other points associated with the same phenotype. As a complementary approach to gauge the robustness of a phenotype to parameter changes, we quantified the Mean Path Length (MPL) as the average number of unit changes or “jumps” in parameter values needed to start from any point within a phenotype subspace and land on a foreign phenotype (Figure 3B, Materials and Methods)[25]. Large values of MPL indicate that many changes in parameter values are needed to change phenotype, signifying a highly robust phenotype.

We calculated the MPL and CS metrics for the 26 phenotypes with the highest PSO, including the wild-type phenotype (Figure 3C). In addition, we computed these metrics for two phenotypes (1°2°2°1°2°1° and 2°1°2°1°2°1°) that occupy less parameter space (ranked 78^th^ and 79^th^, respectively, in terms of PSO, Figure 2D) but are well-established experimental outcomes. Among these 28 phenotypes, our calculations show a high correlation between MPL and CS, suggesting that these two metrics are equivalent ways to gauge the robustness of a phenotype to parameter variations. The model predicts seven phenotypes with CS and MPL values greater than that of wild type. All seven are experimentally observed in *C. elegans*, suggesting that robustness, as quantified by these metrics, may be an important determinant of experimental realizability. Meanwhile, there are 20 phenotypes with CS/MPL metrics lower than the wild type. Among these 20, ten have been observed in *C. elegans* genetics experiments, while the remaining 10 are the aforementioned novel phenotypes that have not been observed in *C. elegans*. Notably, the CS and MPL values of some of these novel phenotypes (e.g., 2°2°2°3°2°2°, 3°2°2°3°2°2°, and 3°2°3°1°3°2°) falls within the range of experimentally observed counterparts, suggesting that these novel phenotypes may be the most realizable experimentally upon performing the correct manipulations in the LIN-3/MAP kinase and the LIN-12 pathways.

### Identifying optimal molecular perturbations to render specific mutant phenotypes

Having predicted novel phenotypes and the experimental realizability of these outcomes, a key question is how does one render such phenotypes experimentally? The classical computational approach is to choose reference parameter values for the wild-type phenotype and then to test the effect of specific parameter perturbations. The choice of parameter perturbation is motivated typically by a corresponding mutation that has been performed experimentally with the goal of determining whether the predicted phenotype matches the experimental outcome. The pitfall, however, is that suitable reference parameter values for the *in vivo* biochemistry of signaling pathways in live worms are unknown. Furthermore, worms are not quantitative clones, and each worm is likely to differ in its parametric settings. Finally, the execution of a particular experimental perturbation is unlikely to be realized in the same quantitative manner in each worm in every trial.

**Figure 4:**
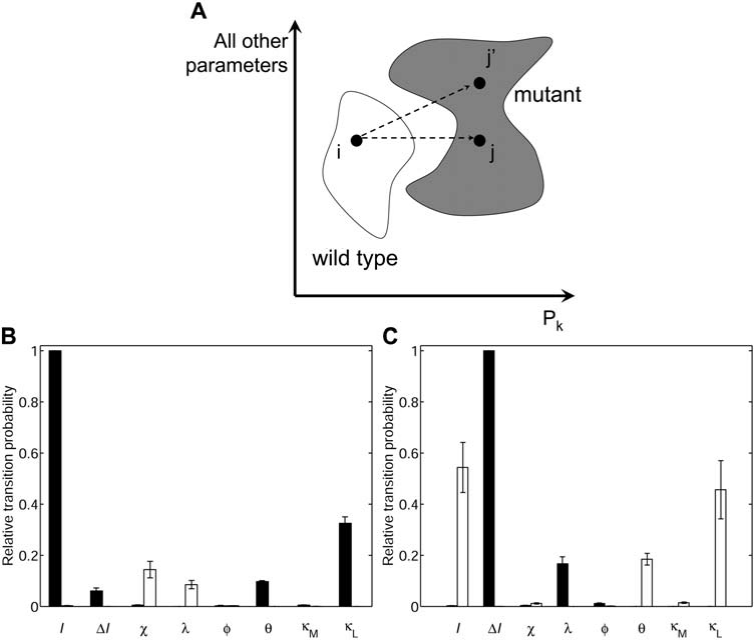
Quantitatively predicting the optimal molecular perturbations needed to generate specific mutant phenotypes. (A) Schematic for counting phenotype transitions made possible by single mutations. Subspaces in the 8-dimensional parameter space are occupied by different phenotypes. This diagram portrays a simplified version of the phenotype phase diagram with a single parameter *P_k_* isolated on the *x*-axis and all other parameters denoted on the *y*-axis. The transition from i→j can occur by a change in a single parameter *P_k_*, but the transition i→j′ cannot. By counting the number of successful single mutations (i→j) for each parameter *P_k_*, we quantify the relative efficacy of each parameter to render a specific phenotype transition (W→M) (see also Materials and Methods). (B,C) The relative probability of inducing a transition from the wild-type phenotype to the 3°3°3°3°3°3° phenotype (B) or the the hyperinduced 2°1°2°1°2°1° phenotype (C) by decreasing (filled columns) or increasing (open columns) the values of parameters indicated on the *x*-axis. The *y*-axes report the mean relative transition probability averaged over a broad combination of threshold values for fate-determining signals, and the error bars denote the standard deviation (see also Materials and Methods). The size of the error bar reveals that model predictions are robust to variations in the threshold values of fate-determining signals.

Based on these considerations, we take a different approach that is enabled by the phase diagram of phenotypes that we have computed for this system. Using this phase diagram, we determine all possible single-parameter changes (i.e., single mutations) that successfully transition the wild-type phenotype into a mutant phenotype of interest. The fraction of these successful single-parameter changes that is associated with a particular parameter reveals the relative efficacy with which that parameter perturbation “transitions” the wild-type phenotype into the mutant outcome (Figure 4A and Materials and Methods). In this manner, these computations yield a transition probability that an increase (or decrease) in each parameter will shift the phenotype from wild type to a mutant pattern. Parameter changes with a higher transition probability have a greater likelihood of generating the desired mutant phenotype. Thus, this approach is the computational equivalent of a random genetic screen that evaluates all possible mutations to determine the most effective ones that lead to the mutant phenotype of interest.

To test initially this approach, we applied it to mutant phenotypes that have been well established by genetics experiments in *C. elegans*. We first predicted the best single-parameter changes needed to transform the wild-type organism into a vulvaless mutant. Vulvaless phenotypes have been observed in genetics experiments and occur when all vulval precursor cells acquire the 3° fate [2],[24],[26]. Our model predicts that the best way to render the 3°3°3°3°3°3° phenotype is by decreasing the level of inductive signaling (Figure 4B). This prediction is consistent with experiments in which anchor cell ablation yields the uninduced all-3° fate pattern [27].

In the other extreme of phenotypes, mutant worms with multiple vulvae have been observed when the inductive signaling pathway is hyperactivated [28]–[30]. In these mutants, the vulval precursor cells acquire an intriguing alternating pattern of 2°1°2°1°2°1° where each 1° cell produces an invagination [31]. Consistent with this experimental observation, the model predicts an increase in inductive signal as one of the most prominent ways to yield this alternating phenotype (Figure 4C).

In addition, because all possible single mutations are evaluated, our model analysis predicts additional “equivalent mutations” that would render the same 2°1°2°1°2°1° phenotypic outcome (Figure 4C). One of these equivalent mutations is to flatten the gradient in soluble inductive factor (Figure 4C). This particular prediction is remarkably consistent with what has been recently uncovered about the most classical experimental mutation to yield this phenotype. The loss of *lin-15* has been shown to cause the secretion of LIN-3 from the surrounding cells, an event that would ablate the gradient [32]. A second equivalent mutation predicted by the model is an increase in the threshold of lateral signaling needed to inhibit the MAP kinase pathway (κ_L_). This prediction for generating a well-established phenotype through a non-canonical perturbation is testable experimentally by decreasing the binding affinity of the lateral signaling transcription complex (LAG-1:LIN-12-cyto) to LBS elements in the *cis*-regulatory regions of the genes that negatively regulate inductive signaling (*ark-1*, *lip-1*, *lst-1,2,3,4*) [33]. This mutation would require greater lateral signaling to inhibit the inductive MAP kinase pathway and would be an indirect way to inflate the inductive signaling activity, conceptually consistent with the direct hyperactivation of the inductive signaling pathway.

An intriguing feature of mutants, such as *lin-15(lf)* [24],[31] and *let-60(gf)* [34], is that the observed multicellular pattern is variable. In addition to 2°1°2°1°2°1°, the other prominent outcome is 1°2°2°1°2°1°. There are several possible sources of variability [5]. The quantitative levels and interactions of signaling molecules may differ among wild-type organisms in which the mutation is performed; thus, their response to a specific perturbation may produce different outcomes. Alternatively, even if two organisms were “quantitative clones,” the magnitude of a perturbation being introduced by the mutation may vary; for example, the amount of RNAi delivered may be different. Finally, even if the perturbation and the wild-type organisms were exactly the same, the execution of the molecular network may deviate due to stochastic effects.

**Figure 5:**
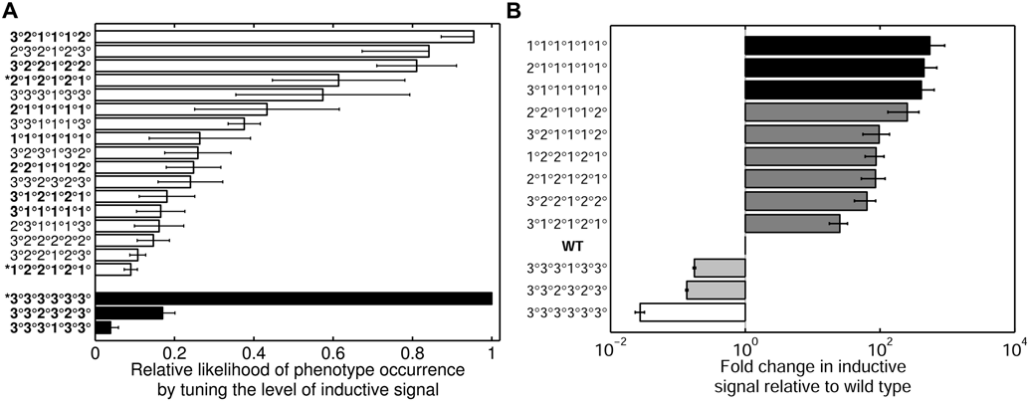
The hierarchy of phenotypes predicted to occur through quantitative changes in morphogen level. (A) The relative probability (*x*-axis) of reaching different mutant phenotypes (*y*-axis) upon decreasing (filled bars) or increasing (open bars) the amount of LIN-3 morphogen. The predicted phenotypes denoted with an asterisk are ones observed experimentally in *C. elegans*. Bold-faced phenotypes correspond to multicellular patterns observed experimentally in the three major species of the *Caenorhabditis* genus (see Figure 6A). (B) The predicted fold change in inductive signal (*x*-axis) necessary to convert the wild-type phenotype into underinduced phenotypes (Fold change <1) and over-induced phenotypes (Fold change >1). The phenotypes listed on the *y*-axis correspond to the bold-faced phenotypes in panel (A). The list has been re-sorted according to the required fold change in inductive signal. The shading of bars corresponds to the shading of experimentally observed phenotypes in Figure 6A.

Regardless of the source of variability, the key question we focused on is why this variability would produce these two particular outcomes and not others. We hypothesized that in the parameter space, variable mutant phenotypes may lie in the same general direction from the wild-type phenotype. That is, because the starting point, the extent of perturbation and the execution of a perturbation may differ (Figure 4A), the target points in parameter space on which these perturbations land will vary but lie within a common vicinity. To test this hypothesis, we determined what other phenotypes would be predicted by the model upon increasing the inductive signal (Figure 5A) or flattening the gradient (Figure S3). Indeed, the 1°2°2°1°2°1° phenotype is predicted to occur in response to both perturbations, revealing that the variable mutant phenotypes lie in the same direction in parameter space from the wild-type phenotype. Furthermore, our model predicts that converting the wild-type phenotype to either the 2°1°2°1°2°1° or 1°2°2°1°2°1° phenotypes would require approximately the same amount of increase in inductive signal (Figure 5B). These predictions confirm the hypothesis that these two phenotypes may co-occur because these outcomes exist at similar positions relative to the wild-type phenotype in the multidimensional parameter space.

### Model-based testing of the quantitative diversification hypothesis

**Figure 6:**
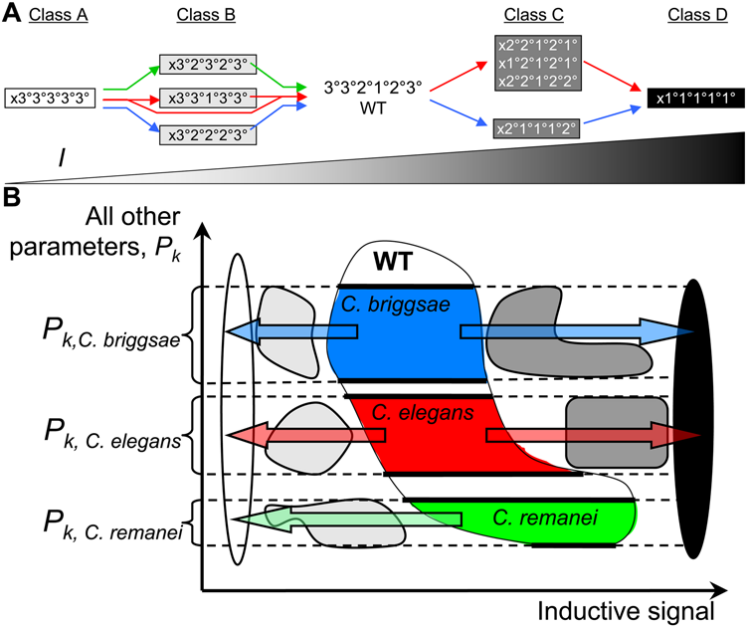
(A) Summary of experimentally observed phenotypes in three different species of the *Caenorhabditis* genus. Distinct phenotypes have been reported upon increasing or decreasing the level of inductive signal (*I*) in three major members of the *Caenorhabditis* genus [22]. The wild-type phenotype (center) is common to all three species. The colored lines denote the species-specific progression of phenotypes as the level of inductive signal (*I*) is modulated in *C. elegans* (red), *C. briggsae* (blue) and *C. remanei* (green). Phenotypes were not reported in *C. remanei* upon increasing inductive signal above the wild-type level [22]; therefore, green arrows are not drawn to the right of the wild-type phenotype. The phenotypes are grouped into Classes A, B, C, and D and are shaded (white, light grey, dark grey and black) according to the amount of perturbation in inductive signal that rendered each mutant. In some species the first pre-cursor cell (P3.p) is not competent to participate in vulval development, and therefore, it is designated as ‘x’. (B) The quantitative diversification hypothesis. Species belonging to the *Caenorhabditis* genus all produce a common wild-type phenotype using a common regulatory network that performs with quantitative differences in parameter settings. Thus, *C. elegans* (red), *C. briggsae* (blue) and *C. remanei* (green) are hypothesized to occupy different subspaces within the wild-type parameter space. This quantitative diversification has been proposed to explain the fact that changes in the level of inductive signal produce species-specific mutant phenotypes.

An apparent conundrum in our model predictions is that when inductive signal is increased, the number of predicted phenotypes is far greater than that observed experimentally in *C. elegans* (Figure 5A). In fact, similar calculations show that phenotypes other than 3°3°3°3°3°3° are possible when the level of inductive signal is decreased (Figure 5A). Why then is the remarkably rich set of predicted phenotypes vastly under sampled in experiments with *C. elegans*? One possibility is that our model predicts phenotypes that may occur when the inductive signal is tuned to intermediate levels; such phenotypes may not be sampled by classical genetics experiments that typically involve knock-out or strong overexpression strategies. Another hypothesis is that our model predicts phenotypes that arise not only in *C. elegans*, but also in several closely related species. Several members of the *Caenorhabditis* genus undertake a similar step in vulval development where precursor cells commit to a 3°3°2°1°2°3° wild type pattern [22],[35],[36]. Compelling recent experiments have revealed that tuning the level of inductive signal produces distinct species-specific mutants even though the starting wild-type phenotype is the same (Figure 6A) [22]. Since vulval development in all of these species involves the same regulatory “parts” (EGF and Notch signaling), these experimental results have raised the intriguing hypothesis that a common regulatory network has quantitatively diversified, so that the network still produces the wild-type phenotype, but when quantitatively perturbed, each species has access to unique phenotypes (Figure 6B).

Experimentally testing this hypothesis of quantitative diversification would involve uncovering the regulatory network driving vulval development in each member of the *Caenorhabditis* genus and proving that the network architecture is indeed the same. This approach raises practical hurdles of performing numerous genetics experiments across multiple species. A deeper challenge is that it is difficult to prove unequivocally that the regulatory network is the same between two species, since one cannot rule out the existence of an undiscovered mechanism. On the other hand, a modeling framework can be particularly effective in testing the quantitative diversification hypothesis. A model can directly test whether the proposed vulval regulatory network that has been inferred from studies in *C. elegans* is capable of rendering the breadth of phenotypes observed across multiple species solely through quantitative changes in regulatory mechanisms.

To conduct this analysis, we compared our predicted phenotypes (Figure 5A) to the experimentally observed phenotypes in the three major members of the *Caenorhabditis* genus, *C. elegans*, *C. briggsae* and *C. remanei* (Figure 6A). We find that 8 of the 9 experimentally observed phenotypes across the three species are captured by our model predictions. These results demonstrate unequivocally that a common vulval developmental network is capable of producing a significant fraction of the phenotypic diversity observed in the three major members of the *Caenorhabditis* genus. Thus, the model provides new and strong support for quantitative diversification of a common vulval developmental network during the evolution of the *Caenorhabditis* genus.

Where the model fails also provides intriguing insight. Our model does not predict the 3°2°2°2°3° phenotype that occurs when inductive signal is decreased moderately in *C. briggsae*. *C. briggsae* is phylogenetically closer to *C. remanei* than to *C. elegans* [22],[36], suggesting that quantitative diversification hypothesis may fail to explain fully how *C. briggsae* and *C. elegans* vulval regulatory networks have diverged. In addition, the model predicts several phenotypes that are not found in *C. elegans*, *C. briggsae* and *C. remanei* (Figure 5A). These additional phenotypes may occur in other members of the *Caenorhabditis* genus. The seminal dataset collected by Felix in fact spans eight additional species. We are currently developing algorithms for systematically clustering and comparing model-predicted phenotypes to this larger experimental dataset.

Meanwhile, an important feature of the experimental data gathered by Felix is that species-specific phenotypes emerge only when the inductive signal is tuned to a certain quantitative level (Figure 6B). The 3°3°3°3°3° (Class A) and 1°1°1°1°1° (Class D) phenotypes are observed only when inductive signal is strongly decreased or increased, respectively; meanwhile Class B (3°2°3°2°3°, 3°2°2°2°3° and 3°3°1°3°3°) and Class C (1°2°1°2°1°, 2°2°1°2°1°, 2°2°1°2°2° and 2°1°1°1°2°) phenotypes occur upon moderate decrease and increase in inductive signal, respectively. Thus, a more rigorous test of quantitative diversification is not only to prove that a common regulatory network can render the breadth of experimentally-observed phenotypes, but also to demonstrate that the predicted phenotypes occur only when the network is tuned in the appropriate quantitative manner. To undertake this more rigorous test of quantitative diversification, we determined the amount of change in inductive signal needed to render the predicted phenotypes. The predicted quantitative hierarchy of phenotypes (Figure 5B) directly matches experimental observations (Figure 6B), providing stronger evidence to support the quantitative diversification hypothesis.

The model predictions directly validate the hypothesis that the parameter space associated with the wild-type phenotype actually contains several subspaces, each representing different species. A key question is which subspace of parameter values corresponds to each species (Figure 6A). The answer to this question would reveal how the quantitative settings of this developmental network have evolved during the emergence of the *Caenorhabditis* genus. To address this question, we analyzed more closely the layout of phenotypes in the multidimensional parameter space. We know that each species produces different phenotypes when the level of inductive signal is changed (Figure 6B) [22]. For example, *C. elegans* transitions from the phenotype 3°2°1°2°3° (WT) to 3°3°1°3°3° when inductive signal is reduced moderately; meanwhile, *C. remanei* forms 3°2°3°2°3° upon intermediate reductions in inductive signal. In both species, a strong reduction in inductive signal produces 3°3°3°3°3°. Therefore, by identifying the subset of wild-type parameter values that produce a WT→3°3°1°3°3°→3°3°3°3°3° transition versus WT→3°2°3°2°3°→3°3°3°3°3°3° transition upon reducing inductive signal, we isolated the *C. elegans* and *C. remanei* parameter subspaces (Materials and Methods). Similarly, *C. briggsae* forms patterns with adjacent 1° fates upon mild increase of inductive morphogen signal, while *C. elegans* requires a strong increase in morphogen activity to render such outcomes. Therefore, by distinguishing between WT→1°2°1°2°1°→1°1°1°1°1° transitions and WT→2°1°1°1°2°→1°1°1°1°1° transitions, we identified the subset of wild-type parameter values that correspond to *C. elegans* and *C. briggsae* subspaces. We find that *C. elegans* represents 41.01±7.90%, *C. briggsae* represents 3.71±1.95%, and *C. remanei* represents 41.31±8.20% of all wild-type space points. The remaining 13.97±2.25% of wild-type space points represents transition patterns that are inconsistent with experimental results for these three species.

**Figure 7:**
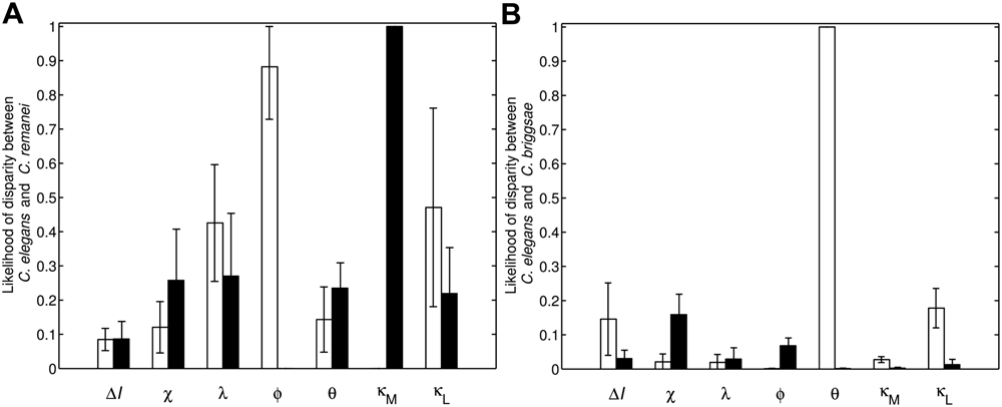
Quantitative differences predicted to have arisen during the evolution of the *Caenorhabditis* genus. The likelihood that *C. remanei* (A) or *C. briggsae* (B) differ from *C. elegans* by higher (open columns) or lower (filled columns) values for parameters indicated on the *x*-axis.

Having identified the sub-region of wild-type parameter space belonging to *C. elegans*, *C. briggsae* and *C. remanei*, we determined how the parameters differ among these species (Figures 7A and 7B). The model identifies two potential groups of parameters. The values of the first group may be higher or lower in *C. elegans* relative to *C. remanei* (*ΔI*, χ, λ, θ and κ_L_) or *C. briggsae* (λ, φ and κ_M_). In contrast, the model predicts that a second group of parameters has changed in a biased manner, either selectively increasing or decreasing, during the evolution of *C. elegans*, *C. remanei*, and *C. briggsae*. The value of φ is higher in *C. remanei* than in *C. elegans*, indicating that inductive signaling produces a stronger lateral signal in *C. remanei* (Figure 7A). Furthermore, the threshold of inductive signaling (κ_M_) needed to trigger the lateral signal is lower in *C. remanei*. Taken together, these predictions reveal that the ability to send out lateral signals is far stronger and more sensitive to inductive signaling in *C. remanei* than in *C. elegans*. On the other hand, *C. briggsae* deviates from *C. elegans* primarily in the ability to receive lateral signals (θ) and thereby inhibit inductive signaling (χ, κ_L_). Inductive signaling is predicted to be more sensitive to lateral inhibition in *C. elegans* than in *C. briggsae* (lower χ and higher κ_L_ in Figure 7B).

These results reveal that during evolution, the members of the *Caenorhabditis* genus have taken remarkably divergent paths in quantitatively modulating a common developmental signaling network. We demonstrate that the underlying quantitative molecular changes can in fact be inferred from experimental observations of phenotypic variability. This inference requires a computational approach, since the underlying molecular signaling network is highly interconnected and its relation to emergent multicellular phenotypes is non-intuitive. Our approach hinges on a mathematical framework for predicting multicellular phenotypes from the underlying signaling network and a broadly applicable computational approach to analyze the phenotypic landscape. With growing interest in quantitative mechanistic models of developmental systems [14],[16],[18], the computational approach described here will likely find broad application in other developmental contexts and offers a systematic approach to mapping the quantitative regulatory changes that have given rise to divergent developmental phenotypes.

## Materials and Methods

### Computational model of *C. elegans* vulval development

#### Signaling network and model equations

The vulva in *C. elegans* and related species develops from a set of equivalent vulva precursor cells (VPCs) labeled Pn.p (n = 3 to 8) in Figure 1 [20]. These cells are arranged linearly along the antero-posterior axis of the body. During the third stage of larval development, the VPCs receive a spatially graded EGF-like stimulus (LIN-3) from the anchor cell (AC) in the gonad. Binding of LIN-3 to its receptor LET-23 activates the MAP kinase MPK-1 and induces their differentiation. In addition to the soluble LIN-3 signal, juxtracrine interactions through Notch-like receptor LIN-12 contribute to VPC differentiation. Together, the inductive LIN-3 signal and the lateral Notch signal establish a pattern of VPC differentiation (3°3°2°1°2°3°) in wild-type organisms. Only the VPCs committed to 1° and 2° fates contribute to vulva formation through cell divisions and spatial rearrangements of the daughter cells; meanwhile, the daughters of the 3°-committed VPC fuse to the hypodermal syncytium.

We previously described a mathematical model of the LIN-3/LIN-12 signaling network [21]. This model was based on the current understanding of the bidirectional coupling between LIN-3 and LIN-12 signaling pathways (Figure S1A). To make the model tractable, we represented multistage signaling cascades and redundant pathways as a single reaction pathway. This coarse-grained representation completely maintains the regulatory logic of the LIN-3/LIN-12 network, while simplifying its mathematical representation. Distinct from other modeling strategies [12],[13], this mathematical model formally encodes the quantitative strength of every molecular interaction in the regulatory network, a necessary feature to probe quantitative diversification.

Ordinary differential equations were formulated to track the level of two fate-encoding signals in each cell *i*: active MAP kinase (MAPK) molecules (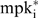) and lateral signal activity (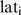). These equations are:
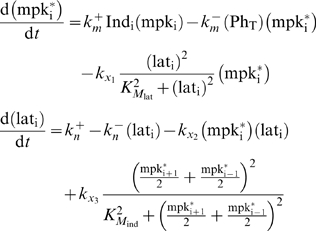
(1)where ν_i_ is the number of neighbors for cell *i* and the other dimensional parameters are described in the legend to Figure S1A.

In addition, each VPC is stimulated by a local amount of inductive signal, Ind_i_. The values for Ind_i_ were determined by modeling diffusive transport of the soluble factor coupled with linear degradation in the extracellular space. At steady state, the gradient is described by:
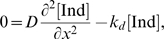
(2)whose solution is:
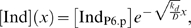
(3)when we require that 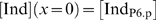. We rewrite this solution by rescaling the spatial axis, *x*, in terms of the length of P3.p-P6.p VPC field, *L*, as follows:
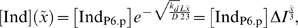
(4)where 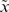 is 0, 1, 2 and 3 for P6.p, P5/7.p, P4/8.p and P3.p, respectively. Thus, the parameters Ind_P6.p_ and *ΔI* specify the local level of inductive signal (Ind_i_). A change in the value of *ΔI* alters the steepness of the exponential gradient in inductive signal.

The dimensional variables 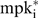 and lat_i_ were normalized by their characteristic values, mpk_T_ and lat_T_, respectively to yield the following nondimensional state variables:
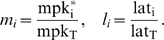
(5)Subsequently, dimensional parameters in the model equations were rearranged to identify the following dimensionless parameter groups:
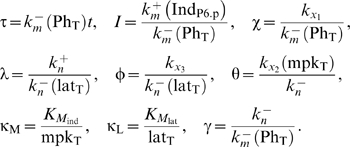
(6) Thus, by using non-dimensional parameters, we have reduced the space of parameters from 13 dimensional parameters to 9 dimensionless ones. This reduction reduces the computational load albeit this load is not prohibitive as others have analyzed parameteric sensitivity of biomolecular networks by sweeping across 36 dimensional parameters[37]. Using these nondimensional quantities, our model equations may be rewritten as follows:
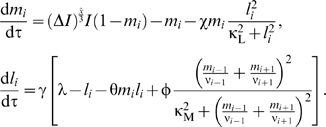
(7)


#### Framework for assigning cell fates

The timing of VPC patterning has been studied by ablating the anchor cell (AC) at different times during the induction process. Results from these experiments have established that the AC (and therefore, the LIN-3 signal that it secretes) is needed for approximately 6 hours in order for the VPCs to commit to the 3°3°2°1°2°3° fate pattern [26],[27]. Our model calculations show that the fate-determining signals (MAP kinase (*m_i_*) and lateral (*l_i_*) signals) reach their steady-state values within 5 hours for reference parameter values (detailed below). Therefore, we worked under the reasonable assumption that the steady-state values of *m_i_* and *l_i_* prescribe the fate choice of each VPC. For all simulations, the steady-state solution of the dimensionless model equations was determined using the initial condition that the levels of inductive and lateral signal are zero in all cells. We note that for steady-state calculations the dimensionless group γ is eliminated from model equations (7).

The output of each simulation is the dimensionless magnitudes of the fate-determining signals (*m_i_*, *l_i_*). These are in turn recast into the dimensional form (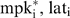) from which fate assignments are determined using the framework that we described previously (Figure S1B) [21]. Briefly, (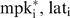) in each VPC is a point in the (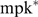, lat) fate plane. Two orthogonal thresholds, (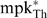, lat_Th_) segregate the fate plane into four quadrants. The dimensional inductive and lateral signals in each cell are compared against their respective threshold values, which then translate into 1°, 2°, 3° or m fate quadrants (Table S1).

### Quantifying phenotypic capacity

In order to explore phenotypes that would result from quantitative variations in network performance, we varied the value of each dimensionless parameter, starting from its central value and expanding in a step-wise fashion by increasing and decreasing its value by ∼3–4 fold. The central values of the dimensionless parameters were determined as described in Supporting Text S1. In this manner, the parameter space hypervolume was expanded sequentially and contained 3^8^, 5^8^, 7^8^, 9^8^ and ultimately 11^8^ points. Therefore, at its maximum size, the parameter space contained 11 values per parameter (equally spaced on a log scale), spanned 5–6 orders of magnitude for each parameter (Tables S2, S3), and represented 11^8^ parameter combinations in total.

For each combination of 8 model parameter values, we computed the fate pattern. Importantly, the fate of each cell *i* is determined by whether the amounts of MAP kinase and lateral signals in that cell (mpk_i_ and lat_i_) exceed threshold levels (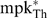 and lat_Th_, respectively; see Table S1). Because these threshold values are unknown, and in fact, may be a source of variation in an evolutionary context, we computed fate patterns across a broad range of threshold values. Specifically, 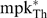 and lat_Th_ were varied across the ranges 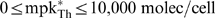 and 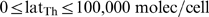, respectively. The cumulative number of fates predicted across the 8-dimensional parameter space for every combination of threshold values is reported in Figure 2C.

### Calculating the Parameter Space Occupancy (PSO)

To quantify the PSO for each phenotype, we determined the number of parameter points associated with each phenotype at every combination of threshold values. This total level of occurrence of each phenotype was divided by the total number of parameter points to yield the fraction of parameter space occupied by that particular phenotype. Phenotypes were binned according to the fraction of parameter space occupied in unit log_10_ bins (i.e., 1 to 0.1, 0.1 to 0.01, etc). The number of distinct phenotypes in each bin is plotted on the *y*-axis in Figure 2D. The distribution of parameter space occupancy was then fit to a log-normal probability distribution. There are 19 phenotypes two standard deviations below the mean (Table S4) and 34 phenotypes two standard deviations above the mean (Table S5).

### Quantifying the robustness of the phenotype subspaces to parameter variations: the Connectivity and Shape (CS) and the Mean Path Length (MPL) metrics

Each point in the 8-dimensional parameter space maps to a phenotype (Figure 2B). We refer to the collection of points in the parameter space that are associated with a particular phenotype as the phenotype subspace. To quantify the CS value for each phenotype, we distinguished between isolated, edge, and interior points in the phenotype subspace. Isolated points are those points for which unit jumps along *both* (increase and decrease) directions of *every* parameter axis lead to points associated with another phenotype. In the other extreme, there are interior points for which unit jumps in *both* directions along *every* parameter axis reach points that still belongs to the same phenotype. Finally, between these possibilities are edge points: a unit jump in *at least one* direction along *at least one* parameter axis leads to another phenotype. To calculate the CS metric for a phenotype, we assign each point in the phenotype subspace a score equal to the number of neighboring points that belong to the same phenotype. This score ranges between 0 (for isolated points) and 16 (for interior points). We add the scores of each point in the phenotype subspace and normalize this total by the maximum possible score for the phenotype space, accounting for edge effects due to finite parameter domains. This normalized score is the CS value plotted in Figure 3C.

A complementary approach to gauge robustness is to quantify how easy it is to drift out of the phenotype subspace by computing the MPL of escape from the phenotype subspace. We choose randomly a point in the subspace and then make unit jumps along a randomly selected parameter axis and direction. We record the number of jumps taken before exiting the phenotype. This process is repeated until the running average number of jumps stabilizes. We conduct 10 such drift trial reseeding the random number generator between trials. The mean path length is the average over these 10 trials.

Importantly, the 8-dimensional phenotype phase diagram will be sensitive to the threshold values of MAPK (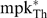) and lateral (lat_Th_) signals. Recall that these thresholds determine how fates are assigned (Table S1). Hence, we computed the MPL and CS metrics across 25 different threshold combinations spanning the following ranges:
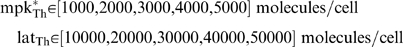

Figure 3C reports the average and standard error across these 25 threshold combinations.

### Predicting the most effective molecular perturbations for rendering mutant phenotypes: the transition probability

Each phenotype, including the wild type, occupies a subspace within the 8-dimensional parameter space (Figure 2B). This phase diagram of phenotypes was analyzed to address the following question: given a choice of 8 single mutations (i.e., 8 parameter perturbations), which single-parameter change (i.e., single mutation) would be most likely to promote a transition from wild-type (W) to a mutant (M) phenotype? To address this question, we rank ordered the parameters according to their relative transition probabilities (Figures 4B and 4C), computed as described below. The same transition probability metric is computed to quantify the single-parameter differences that distinguish *C. elegans* from closely related species (Figures 7A and 7B). For this analysis, “transitions” between parameter spaces associated with *C. elegans* and another species (*C. briggsae* or *C. remanei*) were considered.

For the purpose of this discussion, let *P_k_* denote each dimensionless parameter where *k* = 1 to 8. Let *i* denote a point in the W parameter space, and *j* denote a point in the M-parameter space (Figure 4A). By scanning through all (i, j) pairs, we determined the total number that differ only by a single parameter value. These pairs represent the cases where a single-parameter change can cause a W→M phenotype transition. Among this total number of single-mutation pairs, we determined the fraction of phenotype transitions that are attributable to an increase in a particular parameter *P_k_*. This fraction is the transition probability of W→M phenotype transition by *increasing P_k_*. The same calculation was conducted for quantifying the transition probability due to a decrease in *P_k_*.

To determine the robustness of the transition probability to variations in the fate-determining thresholds, we computed the transition probability for 25 different threshold combinations presented above. Hence, the *y*-axes of Figures 4B, 4C, 7A, and 7B report the mean transition probability computed over all these 25 threshold combinations, and the error bar denotes the standard deviation.

### Predicting the phenotypes accessible through quantitative changes in the level of inductive signal

Starting from the wild-type phenotype, we determined all the mutant phenotypes that may be rendered solely by increasing (or decreasing) the inductive signal. Since some mutant phenotypes are more prevalent than others, we quantified the likelihood that an increase (or decrease) in inductive signal would produce each mutant (M). To quantify this likelihood of phenotype occurrence, we first tallied the total number of ways that a change in inductive signal (*I*) would abolish the wild-type (W) phenotype. Among this total, we quantified the fraction that shifted W to a specific mutant M upon an increase (or decrease) in *I*. This fraction represents the likelihood of producing M phenotype by an increase (or decrease) in inductive signal (*I*).

Phenotype assignments must be sensitive to fate-determining threshold values of MAPK and lateral signals (Table S1). To quantify the robustness of the likelihood of phenotype occurrence to threshold variations, we performed the calculation for 25 different threshold combinations (as described above). The mean of the likelihood of phenotype occurrence is reported in Figure 5A and Figure S2A, and the error bars denote the standard deviation. Figure 5A shows the mutant phenotypes with the greatest likelihood of phenotype occurrence upon an increase (empty) or decrease (filled) in inductive signal. The more complete set of phenotypes, including the ones that occur less frequently, are shown in Figure S2A. Similar calculations were performed to determine the phenotype diversity due to changes in gradient steepness. Figure S3 shows the mutant phenotypes with greatest likelihood of phenotype occurrence upon an increase (empty) and decrease (filled) in gradient steepness. Note the occurrence of 1°2°2°1°2°1° and 2°1°2°1°2°1° phenotypes in both Figure 5A and Figure S3.

In addition to the likelihood of generating a particular mutant phenotype, it is also important to gauge the amount of change in inductive signal needed to render each mutant. Some mutant phenotypes may require only small changes, while others may require substantial perturbations. Therefore, we quantified the fold change in *I* needed to produce a specific mutant phenotype (M). For every increase (or decrease) in *I* that produced phenotype M, we kept track of the associated magnitude of change in *I*. The geometric mean of these magnitudes was computed to give the fold change in *I*. As with other calculations, we examined the robustness of this quantity to variations in fate-determining thresholds. The mean fold change in *I* across a broad range of threshold settings is reported in Figure 5B and Figure S2B, and the error bars represent the standard deviation.

### Partitioning the wild-type subspace into species-specific regions

A key experimental observation is that changes in inductive signal produce species-specific phenotypes [22]. Figure S4 highlights the progression of phenotypes observed in *C. elegans*, *C. briggsae*, and *C. remanei* along the inductive signal axis. We developed a computational approach to analyze how these experimental phenotypes are arranged in our predicted phase diagram of phenotypes with the goal of identifying the regions within the wild-type subspace that belongs to each species.

First, we designated each phenotype with a letter code (Figure S4), so that a string of characters or a word may be used to represent the phenotype progression of each species. Phenotypes that are not described in Figure S4 were designated ‘O’. For example, following the lines for *C. elegans* in Figure S4, one word is APWRD. Using this nomenclature, we identified the words that are consistent with the fate progression observed experimentally in *C. elegans*, *C. briggsae*, and *C. remanei* (Table S6).

Next, we determined the word associated with every predicted point in the wild-type subspace. To construct the word, we varied the value of *I* from its minimum to maximum while holding all other parameter values constant. As the *I*-axis was traversed, we recorded each phenotype with its character designation, thereby yielding a 11-character word (11 characters because of the discretization of the *I*-axis). The length of these words was then condensed by eliminating adjacent repeats of a character. For example, APPPOWOSSDD would become APOWOSD (Figure S5). Since ‘O’ phenotypes include cases where VPCs are designated as ‘m’ fate (a fate whose experimental equivalent remains to be elucidated), we removed ‘O’ from the predicted words. In the example, APOWOSD would become APWSD. Thus, at the end of this step, every point in the wild-type parameter is associated with a word that characterizes how the phenotype would change when *I* is increased or decreased.

Finally, we compared the predicted words associated with each point in wild-type parameter space with the experimentally observed phenotype progressions/words of *C. elegans*, *C. briggsae*, and *C. remanei*. In this manner, we identified the regions within the wild-type parameter space associated with each species.

## Supporting Information

Text S1 Rationale for the central values of dimensionless parameters
(0.32 MB PDF)

Figure S1 Model schematic of regulatory network and fate assignments
(0.43 MB PDF)

Figure S2 Extended set of phenotypes that occur upon changing the level of inductive signal
(0.38 MB PDF)

Figure S3 Phenotypic diversity caused by quantitative changes in gradient steepness
(0.09 MB PDF)

Figure S4 Letter representations of the phenotypes observed in C. elegans, C. briggsae and C. remanei
(0.29 MB PDF)

Figure S5 An illustration of our word representation for the order of phenotypes that occurs as inductive signal is increased
(0.10 MB PDF)

Table S1 Fate assignment based on threshold values
(0.08 MB PDF)

Table S2 The values of dimensional parameters used to determine the center values for the dimensionless parameters
(0.18 MB PDF)

Table S3 Range of values for dimensionless model parameters
(0.06 MB PDF)

Table S4 List of phenotypes with PSO values that are two standard deviations below the mean
(0.04 MB PDF)

Table S5 List of phenotypes with PSO values that are two standard deviations above the mean
(0.04 MB PDF)

Table S6 Characteristic words associated with each species
(0.05 MB PDF)
