# Supplementary material for: Predicting Phenotypic Diversity and the Underlying Quantitative Molecular Transitions
Source: PLoS Comput Biol. 2009 Apr 10;5(4):e1000354. doi: 10.1371/journal.pcbi.1000354 (PMC2661366; doi:10.1371/journal.pcbi.1000354)
Supplement: Text S1 — Rationale for the central values of dimensionless parameters (0.32 MB PDF) [file pcbi.1000354.s001.pdf]

**Text S1 – Rationale for the central values of dimensionless parameters**

*Dimensional model parameters.* Our model involves three types of parameters: those driving protein degradation, those driving gene expression, and those driving bimolecular interactions. For the purpose of converting molar concentrations to number of molecules per cell, we assume a cellular volume of $10^{-12}$ L.

$k_n^-$ is the rate of constitutive degradation of lateral signaling. Lateral signaling is mediated by complexes of LIN-12-cyto with LAG-1 transcription factor. Silencing of lateral signaling occurs due to proteosomal degradation of LIN-12-cyto [1]. The mean half-life of proteins susceptible to proteolysis has been measured to be approximately 43 minutes [2]. Therefore, the reference value of parameter $k_n^-$ was set to 0.016 $\text{min}^{-1}$.

$k_{x_2}$ is the rate constant of lateral signaling destruction due to MPK-1$^*$-mediated endocytosis of LIN-12 receptors. The reference value of $k_{x_2}$ was set to $2\times10^{-6}$ (molec/cell)$^{-1}$ $\text{min}^{-1}$, obtained from the rate constant for receptor/complex endocytosis [3] adjusted for the fraction of active MPK-1$^*$.

$k_n^+$ is the constitutive rate of lateral signaling synthesis. This process it thought to be limited by generation of LIN-12 receptors or their ligands. We set the reference value for $k_n^+$ at 130 molec/cell/min based on the rate of EGF receptor (EGFR) synthesis [3].

$k_{x_3}$ is the rate constant for MPK-1$^*$ induced lateral signal generation through transcription of LIN-12 ligands. $k_{x_3}$ reference value is set at 1300 molec/cell/min, which is one order of magnitude larger than the constitutive rates of lateral signal synthesis $k_n^+$. This latter estimate is based on tenfold change in promoter activity over the basal value [4,5].

$k_m^+$ and $k_m^-$ are bimolecular rate constants describing the activation and deactivation of MAP kinase MPK-1. Their reference value was estimated from $k_{cat}/K_M$ values for activation and deactivation of MAP kinase by upstream kinases and phosphatases, respectively [6-8]. Hence, the reference value for these parameters is $2\times10^{-4}$ (molec/cell)$^{-1}$ $\text{min}^{-1}$.

The coupling term $k_{x_1}$ combines protein production by lateral signaling induced gene expression and bimolecular reaction between the phosphatase gene products and active MPK-1$^*$. Hence, the reference value is 2 $\text{min}^{-1}$, estimated from the bimolecular reaction rate constant $k_m^-$ and characteristic levels of induced phosphatase gene expression products which we consider to be $10^4$ molecules/cell.

We consider that there is a constant number of constitutive phosphatase molecules deactivating MPK-1$^*$ during the vulva specification process, and we take this number to be

$Ph_T = 5 \times 10^3$ molecules/cell. Furthermore, the total amount of MPK-1, active and inactive, does not change during this event; hence, we set $mpk_T = 10^4$ molecules/cell [6]. The characteristic level of lateral signal $lat_T$ is estimated to be on the order of high cellular protein copy numbers; thus, we take $lat_T = 10^5$ molecules/cell. Occupied cell surface receptor levels vary from 100 to 100000 receptors/cell [3] therefore take the following reference value, $Ind_{P6.p} = 10^4$ molecules/cell. Concentration of transcription factors in the nucleus can range from 300 to 10000 molecules/nucleus [9]. Thus, we set the reference values $K_{M_{Ind}}$ = 1000 molecules/cell and $K_{M_{lat}}$ = 5000 molecules/cell.

*Centerpoint of dimensionless parameter space.* From the reference values for dimensional parameters (Table S2), the following values for dimensionless parameters were computed (using equations (6) of main text):

$$I = 2, \quad \chi = 2, \quad \lambda = 0.08, \quad \phi = 0.8, \quad \theta = 1.25, \quad \kappa_M = 0.1, \quad \kappa_L = 0.05.$$

These values were used as the center point to construct the multidimensional parameter space. To determine a center value for the gradient steepness, we note that a flat gradient would be represented by $\Delta I = 1$. In contrast, the steepest gradient occurs when a maximum number of receptors [3] ($10^5$ molec/cell) are occupied in the P6.p cell and only a single receptor is occupied in the P3.p cell. Thus, the steepest gradient is represented by $\Delta I = 10^{-5}$. The center value for $\Delta I$ (Table S3) was chosen to approximate the geometric mean of these limiting scenarios.

**References**


1. Ilagan MX, Kopan R (2007) SnapShot: notch signaling pathway. Cell 128: 1246.
2. Belle A, Tanay A, Bitincka L, Shamir R, O'Shea EK (2006) Quantification of protein half-lives in the budding yeast proteome. Proc Natl Acad Sci U S A 103: 13004-13009.
3. Lauffenburger DA, Linderman JJ (1993) Receptors : models for binding, trafficking, and signaling. New York: Oxford University Press. x, 365 p.
4. Buchler NE, Gerland U, Hwa T (2005) Nonlinear protein degradation and the function of genetic circuits. Proc Natl Acad Sci U S A 102: 9559-9564.
5. Lutz R, Bujard H (1997) Independent and tight regulation of transcriptional units in Escherichia coli via the LacR/O, the TetR/O and AraC/I1-I2 regulatory elements. Nucleic Acids Res 25: 1203-1210.
6. Asthagiri AR, Lauffenburger DA (2001) A computational study of feedback effects on signal dynamics in a mitogen-activated protein kinase (MAPK) pathway model. Biotechnol Prog 17: 227-239.
7. Force T, Bonventre JV, Heidecker G, Rapp U, Avruch J, et al. (1994) Enzymatic characteristics of the c-Raf-1 protein kinase. Proc Natl Acad Sci U S A 91: 1270-1274.
8. Zhou B, Wang ZX, Zhao Y, Brautigan DL, Zhang ZY (2002) The specificity of extracellular signal-regulated kinase 2 dephosphorylation by protein phosphatases. J Biol Chem 277: 31818-31825.
9. Bolouri H, Davidson EH (2003) Transcriptional regulatory cascades in development: initial rates, not steady state, determine network kinetics. Proc Natl Acad Sci U S A 100: 9371-9376.
