# Supplementary material for: Predicting Phenotypic Diversity and the Underlying Quantitative Molecular Transitions
Source: PLoS Comput Biol. 2009 Apr 10;5(4):e1000354. doi: 10.1371/journal.pcbi.1000354 (PMC2661366; doi:10.1371/journal.pcbi.1000354)
Supplement: Figure S1 — Model schematic of regulatory network and fate assignments (0.43 MB PDF) [file pcbi.1000354.s002.pdf]

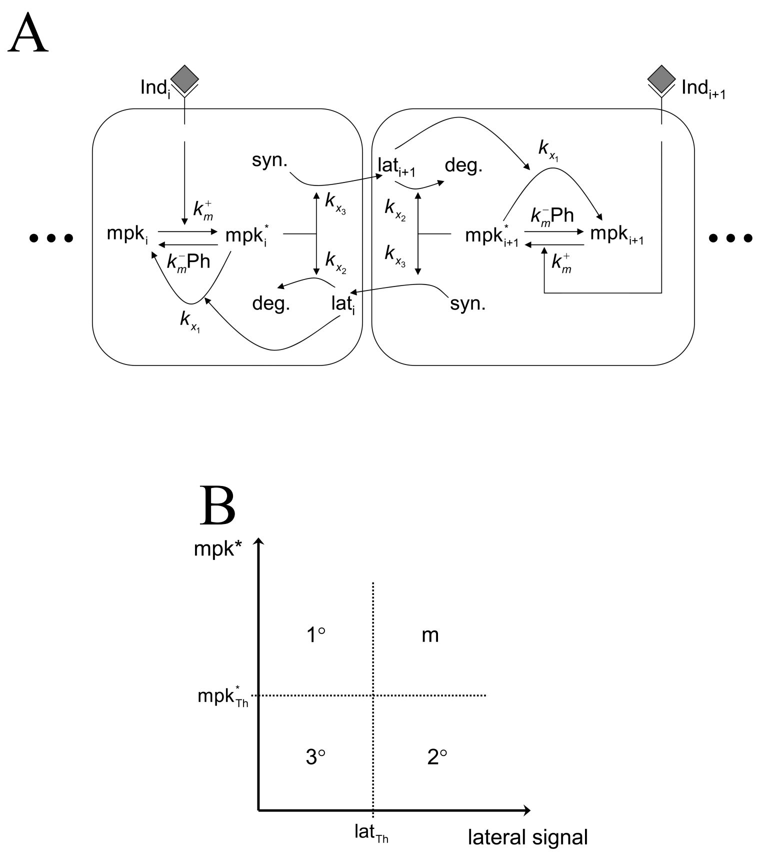

Figure S1. (A) Model schematic of regulatory network governing multicellular patterning during *C. elegans* vulval development. A pair of cells, *i* and *i+1*, within the VPC array are shown; in each cell *i* the inductive LIN-3 signal (Ind_i) activates MPK-1 in each cell with rate constant k_m^+. The deactivation of MPK-1* occurs at a rate determined by the expression level of constitutive phosphatases (Ph_T) and the rate constant k_m^-. The lateral signal mediated by receptor LIN-12 is constitutively activated in each cell at rate k_n^+ and degrades linearly with rate constant k_n^- (not depicted). The inductive signal upregulates the lateral signal in the neighboring cell with rate constant k_{x_3} and downregulates it in the same cell with rate constant k_{x_2}. In turn, the lateral signal in each cell deactivates MPK-1* with rate constant k_{x_1}. **(B) The fate plane for assigning cell fates.** Each cell can take one of four fates (1°, 2°, 3°, m) in the model. The fate of a cell is assigned based on the level of mpk* and lateral signal (lat) based on two thresholds (mpk^*_{Th} and lat_{Th}).
