# Supplementary material for: Predicting Phenotypic Diversity and the Underlying Quantitative Molecular Transitions
Source: PLoS Comput Biol. 2009 Apr 10;5(4):e1000354. doi: 10.1371/journal.pcbi.1000354 (PMC2661366; doi:10.1371/journal.pcbi.1000354)
Supplement: Figure S2 — Extended set of phenotypes that occur upon changing the level of inductive signal (0.38 MB PDF) [file pcbi.1000354.s003.pdf]

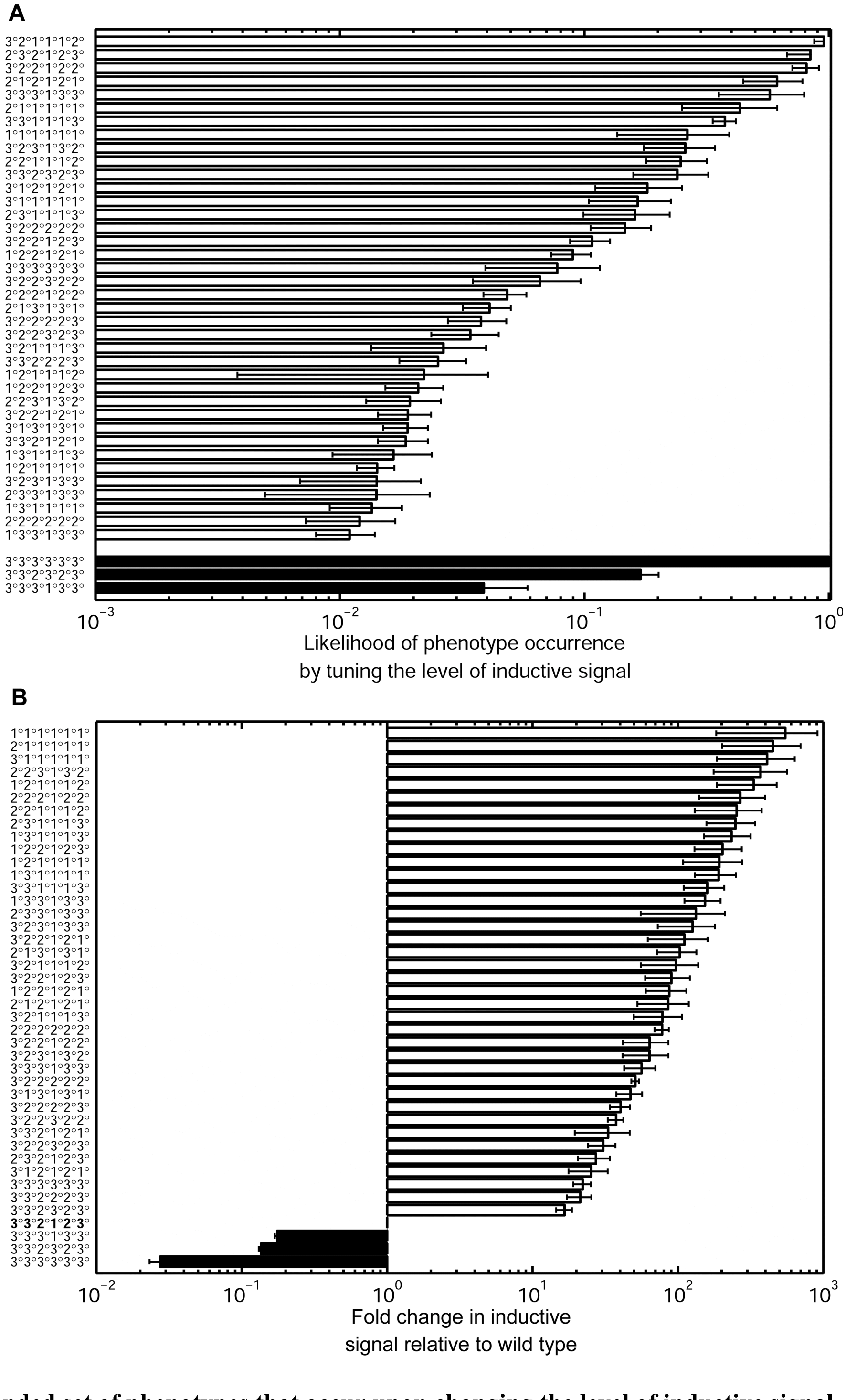

Figure S2. Extended set of phenotypes that occur upon changing the level of inductive signal. (A) Ranking of phenotypes (*y*-axis) according to their likelihood (*x*-axis) to be reached upon increasing (empty) or decreasing (filled) the level of inductive signal (*I*). (B) The fold change in *I* (*x*-axis) required to generate each phenotype (*y*-axis).
