# Supplementary material for: Predicting Phenotypic Diversity and the Underlying Quantitative Molecular Transitions
Source: PLoS Comput Biol. 2009 Apr 10;5(4):e1000354. doi: 10.1371/journal.pcbi.1000354 (PMC2661366; doi:10.1371/journal.pcbi.1000354)
Supplement: Figure S3 — Phenotypic diversity caused by quantitative changes in gradient steepness (0.09 MB PDF) [file pcbi.1000354.s004.pdf]

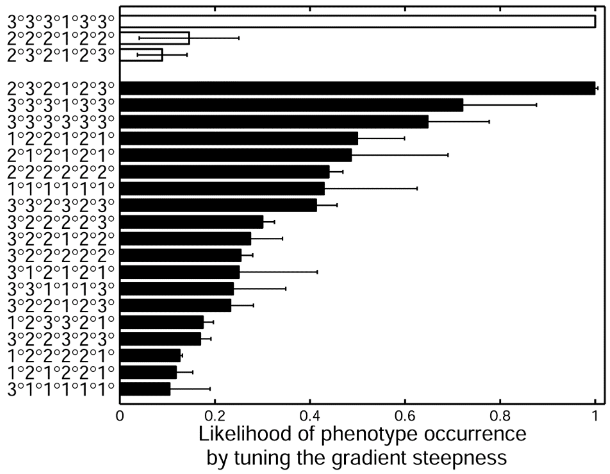


**Figure S3. Phenotypic diversity caused by quantitative changes in gradient steepness.** Mutant phenotypes with greatest likelihood of phenotype occurrence upon an increase (empty) and decrease (filled) in the steepness of the gradient in inductive signal ($\Delta I$).
