# Supplementary material for: Predicting Phenotypic Diversity and the Underlying Quantitative Molecular Transitions
Source: PLoS Comput Biol. 2009 Apr 10;5(4):e1000354. doi: 10.1371/journal.pcbi.1000354 (PMC2661366; doi:10.1371/journal.pcbi.1000354)
Supplement: Figure S4 — Letter representations of the phenotypes observed in C. elegans, C. briggsae and C. remanei (0.29 MB PDF) [file pcbi.1000354.s005.pdf]

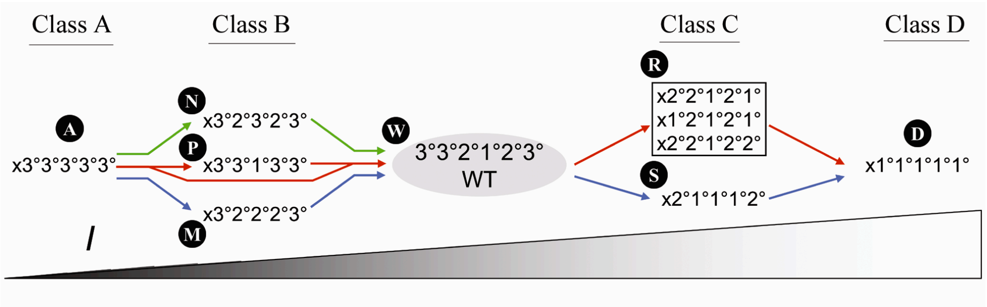

Figure S4. Letter labeling of the phenotypes observed in *C. elegans*, *C. briggsae* and *C. remanei*. Most of this figure is based directly on Figure 6A and summarizes the phenotypes reported in *C. elegans*, *C. briggsae* and *C. remanei* as the level of inductive signal (*I*) is increased (Felix, 2007). Here, we have assigned specific letter labels to the phenotypes. The wild-type phenotype is labeled W. The phenotypes within classes B and C are labeled as N, P, M, R and S in order to distinguish species-specific phenotypes. Classes A, B, C and D are described in the legend to Figure 6A.
