# Supplementary material for: Predicting Phenotypic Diversity and the Underlying Quantitative Molecular Transitions
Source: PLoS Comput Biol. 2009 Apr 10;5(4):e1000354. doi: 10.1371/journal.pcbi.1000354 (PMC2661366; doi:10.1371/journal.pcbi.1000354)
Supplement: Figure S5 — An illustration of our word representation for the order of phenotypes that occurs as inductive signal is increased (0.10 MB PDF) [file pcbi.1000354.s006.pdf]

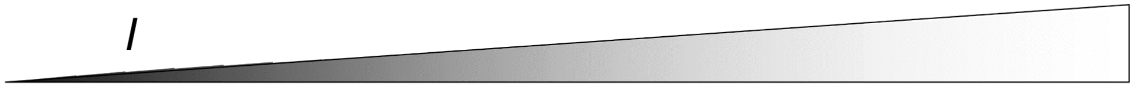

3°3°3°3°3°3°	3°3°3°1°3°3°	3°3°3°1°3°3°	3°3°3°1°3°3°	2°3°2°1°2°3°	3°3°2°1°2°3°	3°2°2°1°2°3°	3°2°1°1°1°2°	3°2°1°1°1°2°	2°1°1°1°1°1°	2°1°1°1°1°1°
A	P			O	W	O	S		D	

Figure S5. An illustration of our word representation for the order of phenotypes that occurs as inductive signal is increased. A sample wild-type (W) space point that leads to 3°3°3°3°3°3°, 3°3°3°1°3°3° and 2°3°2°1°2°3° phenotypes and 3°2°2°1°2°3°, 3°2°1°1°1°2° and 2°1°1°1°1°1° phenotypes upon decreasing and increasing morphogen levels, respectively, is translated to its corresponding word (APOWOSD) according to the procedure detailed in Materials and Methods.
