# Supplementary material for: Predicting Phenotypic Diversity and the Underlying Quantitative Molecular Transitions
Source: PLoS Comput Biol. 2009 Apr 10;5(4):e1000354. doi: 10.1371/journal.pcbi.1000354 (PMC2661366; doi:10.1371/journal.pcbi.1000354)
Supplement: Table S1 — Fate assignment based on threshold values (0.08 MB PDF) [file pcbi.1000354.s007.pdf]

$mpk^*_i \le mpk^*_{Th}$	$lat_i \le lat_{Th}$	Fate
Yes	No	1°
No	Yes	2°
Yes	Yes	3°
No	No	m

Table S1. Fate assignment based on threshold values. For each of the six vulval precursor cells, the dimensional solution to model equations (7) is compared against the thresholds (mpk^*_{Th} , lat_{Th}) to determine its fate choice. This procedure leads to a six-cell pattern of fates. The values of the thresholds are varied to examine the robustness of model predictions to threshold settings (see Materials and Methods).
