# Supplementary material for: Predicting Phenotypic Diversity and the Underlying Quantitative Molecular Transitions
Source: PLoS Comput Biol. 2009 Apr 10;5(4):e1000354. doi: 10.1371/journal.pcbi.1000354 (PMC2661366; doi:10.1371/journal.pcbi.1000354)
Supplement: Table S2 — The values of dimensional parameters used to determine the center values for the dimensionless parameters (0.18 MB PDF) [file pcbi.1000354.s008.pdf]

Parameters	Reference Value	Units
k_m^+, k_m^-	2×10^{-4}	$(\text{molec/cell})^{-1}\ \text{min}^{-1}$
k_{x_2}	2×10^{-6}	$(\text{molec/cell})^{-1}\ \text{min}^{-1}$
k_n^+	130	molec/cell/min
k_{x_3}	1300	molec/cell/min
k_n^-	0.016	min^{-1}
k_{x_1}	2.0	min^{-1}
$K_{M_{\text{Ind}}}$	1000	molec/cell
$K_{M_{\text{lat}}}$	5000	molec/cell
$\text{Ind}_{\text{P6.p}}$	10^4	molec/cell
mpk_T	10^4	molec/cell
lat_T	10^5	molec/cell
Ph_T	5×10^3	molec/cell

Table S2. The values of dimensional parameters used to determine the center values for the dimensionless parameters. These values for the dimensional model parameters were used to compute the central values of the dimensionless parameters around which the parameter space was constructed. The rationale for these parameter values is provided in the Supporting Text.
