# Supplementary material for: Predicting Phenotypic Diversity and the Underlying Quantitative Molecular Transitions
Source: PLoS Comput Biol. 2009 Apr 10;5(4):e1000354. doi: 10.1371/journal.pcbi.1000354 (PMC2661366; doi:10.1371/journal.pcbi.1000354)
Supplement: Table S3 — Range of values for dimensionless model parameters (0.06 MB PDF) [file pcbi.1000354.s009.pdf]

**Dimensionless Parameter**	**Center Value**	**Maximum Value**	**Minimum Value**
I	0.93	5.6×10^{2}	1.5×10^{-3}
ΔI	6×10^{-3}	3.6×10^{-5} (steepest)	1 (most shallow)
χ	0.93	5.6×10^{2}	1.5×10^{-3}
λ	5×10^{-2}	8.0	2.9×10^{-4}
ϕ	0.48	2.9×10^{2}	8.0×10^{-4}
θ	1.61	9.7×10^{2}	2.7×10^{-3}
κ_M	6×10^{-2}	10.0	3.6×10^{-4}
κ_L	4×10^{-2}	6.5	2.32×10^{-4}

Table S3. Range of values for dimensionless model parameters.
