# Supplementary material for: Predicting Phenotypic Diversity and the Underlying Quantitative Molecular Transitions
Source: PLoS Comput Biol. 2009 Apr 10;5(4):e1000354. doi: 10.1371/journal.pcbi.1000354 (PMC2661366; doi:10.1371/journal.pcbi.1000354)
Supplement: Table S4 — List of phenotypes with PSO values that are two standard deviations below the mean (0.04 MB PDF) [file pcbi.1000354.s010.pdf]

Phenotype	Frequency
2° 2° 1° 3° 1° 3°	1.21×10^{-13}
m 2° 1° 1° m 1°	1.05×10^{-13}
m 2° m 1° 2° 2°	8.56×10^{-14}
1° 2° 3° m 2° 2°	7.86×10^{-14}
1° 2° 1° m 2° 2°	6.18×10^{-14}
2° 2° 1° m 2° 1°	4.36×10^{-14}
m 2° 1° m m 2°	3.48×10^{-14}
1° 2° 3° 2° 2° 2°	3.46×10^{-14}
m 2° 1° 1° 1° 1°	1.09×10^{-14}
3° 2° 1° 3° 3° 2°	1.03×10^{-14}

Phenotype	Frequency
2° 2° 1° 3° 3° 2°	5.12×10^{-15}
2° 3° 1° 3° 3° 3°	2.36×10^{-15}
3° 2° m 2° m 2°	$2{\cdot}10\times10^{-15}$
2° 2° 1° 2° 3° 2°	8.45×10^{-16}
3° 2° 1° 2° 3° 2°	7.42×10^{-16}
2° 2° 1° 3° 3° 3°	3.67×10^{-16}
2° 2° 1° 2° m 2°	3.38×10^{-16}
3° m 1° 1° 2° 1°	2.72×10^{-16}
2° 2° 1° 2° 2° 2°	6.21×10^{-18}

Table S4. List of phenotypes with PSO values that are two standard deviations below the mean.
