# Supplementary material for: Predicting Phenotypic Diversity and the Underlying Quantitative Molecular Transitions
Source: PLoS Comput Biol. 2009 Apr 10;5(4):e1000354. doi: 10.1371/journal.pcbi.1000354 (PMC2661366; doi:10.1371/journal.pcbi.1000354)
Supplement: Table S5 — List of phenotypes with PSO values that are two standard deviations above the mean (0.04 MB PDF) [file pcbi.1000354.s011.pdf]

Phenotype	Frequency
3° 3° 3° 3° 3° 3°	0.5458
3° 3° 3° 1° 3° 3°	0.1293
1° 1° 1° 1° 1° 1°	0.0967
3° 3° 1° 1° 1° 3°	0.0949
3° 1° 1° 1° 1° 1°	0.0538
2° 2° 2° 2° 2° 2°	0.0146
3° 3° 2° 3° 2° 3°	0.0109
3° 2° 2° 2° 2° 2°	0.0065
m m m m m m	0.0061
2° 2° 2° m 2° 2°	0.0053
2° 2° m m m 2°	0.0052
2° m m m m m	0.0032
3° 3° 2° 2° 2° 3°	0.0028
3° 2° 2° m 2° 2°	0.0022
3° 3° 2° 1° 2° 3°	0.0020
2° 2° 2° 3° 2° 2°	0.0014
3° 2° 2° 3° 2° 2°	0.0013

Phenotype	Frequency
2° 3° 1° 1° 1° 3°	0.0011
2° 2° 3° 3° 3° 2°	0.0011
2° 2° 3° 1° 3° 2°	0.0011
3° 2° m m m 2°	0.0011
2° 2° 1° 1° 1° 2°	0.0009
2° 3° 3° 1° 3° 3°	0.0009
3° 2° 2° 2° 2° 3°	0.0008
2° 2° 2° 1° 2° 2°	0.0007
2° 3° 3° 3° 3° 3°	0.0007
3° 1° 3° 1° 3° 1°	0.0007
3° 2° 3° 3° 3° 2°	0.0006
3° 2° 3° 1° 3° 2°	0.0006
3° 2° 2° 1° 2° 2°	0.0006
3° 3° 2° m° 2° 3°	0.0006
2° 1° 1° 1° 1° 1°	0.0005
1° m m m m 1°	0.0004
1° 3° 3° 1° 3° 1°	0.0004

Table S5. List of phenotypes with PSO values that are two standard deviations above the mean.
