# Supplementary material for: Predicting Phenotypic Diversity and the Underlying Quantitative Molecular Transitions
Source: PLoS Comput Biol. 2009 Apr 10;5(4):e1000354. doi: 10.1371/journal.pcbi.1000354 (PMC2661366; doi:10.1371/journal.pcbi.1000354)
Supplement: Table S6 — Characteristic words associated with each species (0.05 MB PDF) [file pcbi.1000354.s012.pdf]

Species	Words assigned
C. elegans	AW, APW, AWR, APWR, AWRD, APWRD, WRD, WR, PW, PWRD, PWR
C. briggsae	AMW, AWSD, AWS, APWSD, APWS, WSD, WS, PWSD, PWS
C. remanei	(A)NW*

Table S6. Characteristic words associated with each species. These words are formed from letters designated for each phenotype in order to represent the experimentally observed, species-specific phenotype progressions along the inductive signal (*I*) axis.
